# Hypertrophic obstructive cardiomyopathy complicated by severe midventricular obstruction treated with percutaneous transluminal septal myocardial ablation using simultaneous contrast imaging with left ventriculography and coronary angiography: a case report

**DOI:** 10.1093/ehjcr/ytag659

**Published:** 2026-08-31

**Authors:** Togo Sakai, Masao Takemoto, Kotaro Numaguchi, Takuya Tsuchihashi

**Affiliations:** Cardiovascular Centre, Social Medical Corporation, Steel Memorial Yawata Hospital, Haruno-machi, Yahatahigashi-ku, Kitakyushu 805-8508, Japan; Cardiovascular Centre, Social Medical Corporation, Steel Memorial Yawata Hospital, Haruno-machi, Yahatahigashi-ku, Kitakyushu 805-8508, Japan; Department of Cardiology, Kitakyushu Municipal Medical Center, Kitakyushu 802-8561, Japan; Cardiovascular Centre, Social Medical Corporation, Steel Memorial Yawata Hospital, Haruno-machi, Yahatahigashi-ku, Kitakyushu 805-8508, Japan

**Keywords:** Case report, Coronary angiography, Hypertrophic obstructive cardiomyopathy, Left ventriculography, Midventricular obstruction, Percutaneous transluminal septal myocardial ablation, Simultaneous contrast imaging

## Abstract

**Background:**

Hypertrophic obstructive cardiomyopathy (HOCM) is a genetic cardiac disease characterized by left ventricular hypertrophy that induces left ventricular outflow tract obstruction (LVOTO), which occurs in ∼25% of patients with hypertrophic cardiomyopathy (HCM), and, less commonly, a midventricular obstruction (MVO). Percutaneous transluminal septal myocardial ablation (PTSMA) treatment for HOCM has become an established therapy to alleviate LVOTO.

**Case summary:**

A 78-year-old woman with HOCM and severe MVO underwent PTSMA using simultaneous contrast imaging with left ventriculography (LVG) and coronary angiography (CAG). After the procedure, the pressure gradient between the aorta and left ventricular apex improved from 140 to 35 mmHg at rest and from 195 to 40 mmHg at post-extra-systolic potentiation. The residual pressure gradient in the left ventricular mid-ventricle and left ventricular outflow tract was 15 and 10 mmHg, respectively. Her clinical condition improved significantly, and she remained well without any symptoms.

**Discussion:**

Midventricular obstruction is relatively rare, occurring in ∼5%–10% of patients with hypertrophic cardiomyopathy; therefore, it is substantially underdiagnosed or missed, leading to suboptimal treatment in a high proportion of patients. Thus, physicians should consider the possibility of MVO when evaluating patients with HCM to avoid overlooking it. Confirmation and clarification of the detailed anatomical relationship between MVO/LVOTO and effective septal branches using contrast imaging with LVG, CAG, and occlusion tests might be essential for achieving effective clinical outcomes in patients with HOCM concomitant with MVO.

Learning points

- Pre-and intra-procedural imaging is important to discriminate between patients with and without midventricular obstruction (MVO).
- Physicians should consider the possibility of MVO when encountering patients with hypertrophic cardiomyopathy (HCM) to avoid overlooking them.
- Simultaneous contrast imaging with left ventriculography and coronary angiography may be an optional treatment strategy for patients with HCM and MVO.

## Introduction

Hypertrophic obstructive cardiomyopathy (HOCM) is a genetic cardiac disease characterized by left ventricular (LV) hypertrophy (LVH), which induces LV outflow tract (LVOT) obstruction (LVOTO).^1,2^ Left ventricular outflow tract obstruction occurs in ∼25% of patients with hypertrophic cardiomyopathy (HCM) and, less commonly, in those with midventricular obstruction (MVO).^1,2^ Percutaneous transluminal septal myocardial ablation^3–5^ (PTSMA) for HOCM is an established therapy for alleviating LVOTO.^3–6^

## Summary figure

**Figure:**
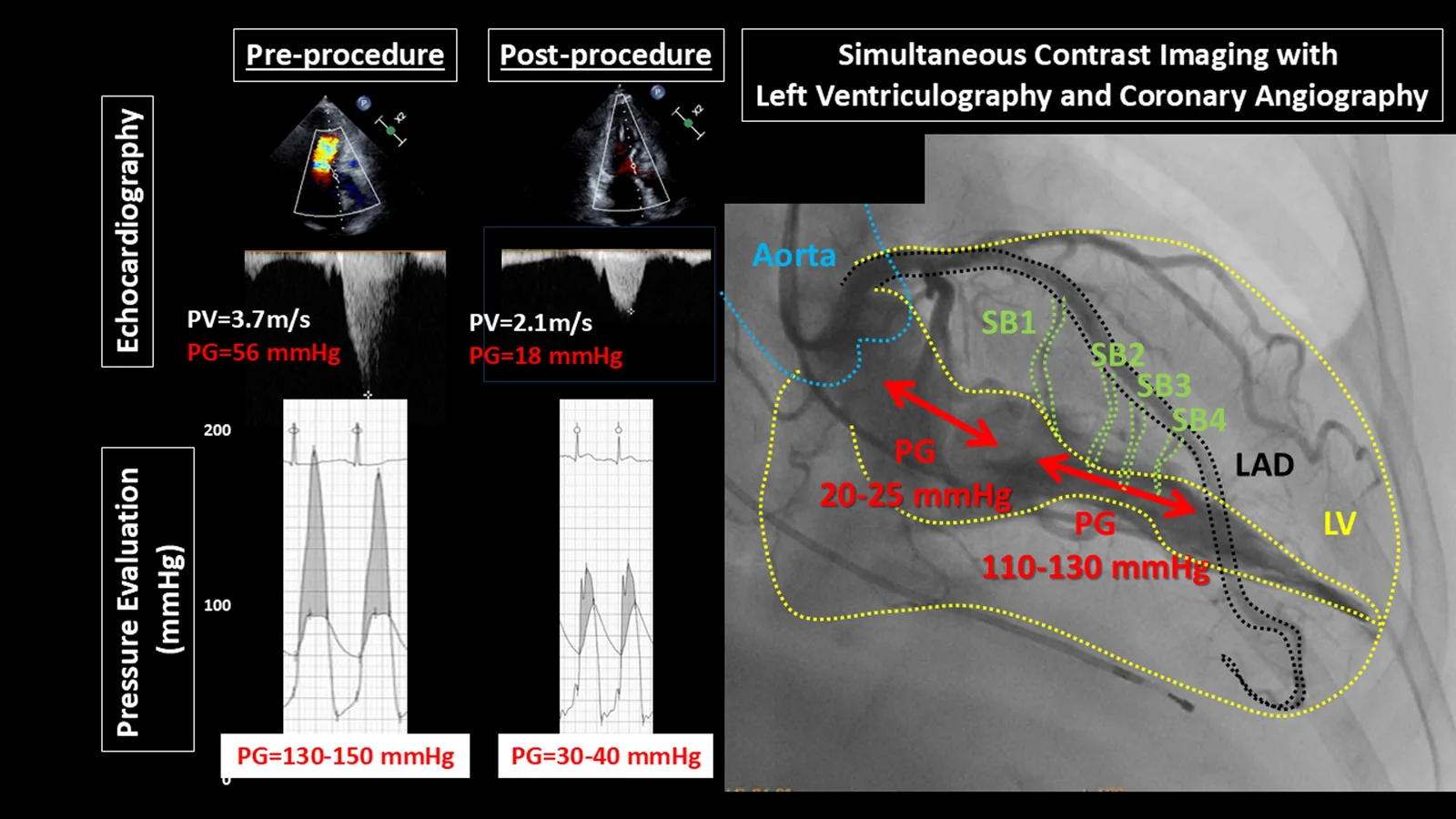
LAD, left anterior descending artery; LV, left ventricle; PG, pressure gradient; PV, peak velocity; SB, septal branch.

## Case presentation

A 78-year-old woman was referred to our hospital with a chief complaint of exertional dyspnoea. The patient had a family history of HCM. She had been receiving treatment for HOCM for several years and for atrial fibrillation (AF) for several months. Her functional status gradually deteriorated to New York Heart Association (NYHA) Class III. Bisoprolol (5 mg) and edoxaban (30 mg) were administered once daily. Disopyramide and verapamil were discontinued due to adverse effects. Mavacamten had not been introduced because of its high cost.

On admission, the patient had a blood pressure of 126/76 mmHg and a regular heart rate of 50 beats/min. Auscultation revealed a systolic murmur (Levine Grade 4/6) at the apex and normal breathing sounds. She had pretibial pitting oedema and a B-type natriuretic peptide (BNP) level of 643.3 pg/mL. A 12-lead electrocardiogram revealed sinus rhythm and LVH with a strain pattern (*Figure 1A*), and chest radiography revealed cardiomegaly (*Figure 1B*). Echocardiography revealed a preserved LV ejection fraction of 71.8%, trivial mitral regurgitation, LVH with an interventricular septal (IVS) thickness of 17 mm, systolic anterior motion (SAM) (*Figure 2A*), and left atrial enlargement with no asymmetric septal hypertrophy or apical aneurysms. Although the sonographer did not detect MVO, colour Doppler imaging revealed turbulent flow in the LV (*Figure 2B*). The maximum pressure gradient (PG) in the LVOT was 48 mmHg at rest and 56 mmHg during the Valsalva manoeuvre (*Figure 2C*). Cardiac computed tomography (CT) revealed massive diffuse LVH and papillary muscle hypertrophy with an IVS thickness of 23 mm (*Figure 1C*). Echocardiography and cardiac CT revealed no evidence of an accessory papillary muscle, elongated anterior mitral leaflet, abnormal chordal insertion, or abnormal position of the papillary muscle. She underwent left ventriculography (LVG), LV catheterization, and coronary angiography (CAG), which revealed LVOT and septal ventricular hypertrophy, an elevated PG between the aorta and LV apex of 130–150 mmHg (*Figure 3A*) at rest and 195 mmHg (*Figure 3B*) at post-extra-systolic potentiation, and additional gradients of ∼110–130 mmHg at the LV mid-cavitary level and 20–25 mmHg at the LVOT (*Figure 3C*), leading to a diagnosis of mild LVOTO and severe MVO. Coronary angiography confirmed the absence of significant coronary stenosis and identified the septal branches (SBs) as PTSMA targets. According to current guidelines,^7,8^ surgical intervention with septal myectomy is the gold standard treatment (Class I), particularly for young patients. Moreover, a recent report has demonstrated the efficacy and safety of septal myectomy for MVO.^9^ However, in the present case, the patient refused open-heart surgery^10^ due to her advanced age (78 years). On the other hand, the patient declined pacemaker implantation^11^ as a primary therapeutic option but accepted it as a bailout treatment. Finally, our cardiology team concluded that PTSMA could be considered because she was older than 65 years of age, had no signs of valvular disease, and had an LV wall thickness of <30 mm.^12^ After providing informed consent, the patient underwent PTSMA.

**Figure 1:**
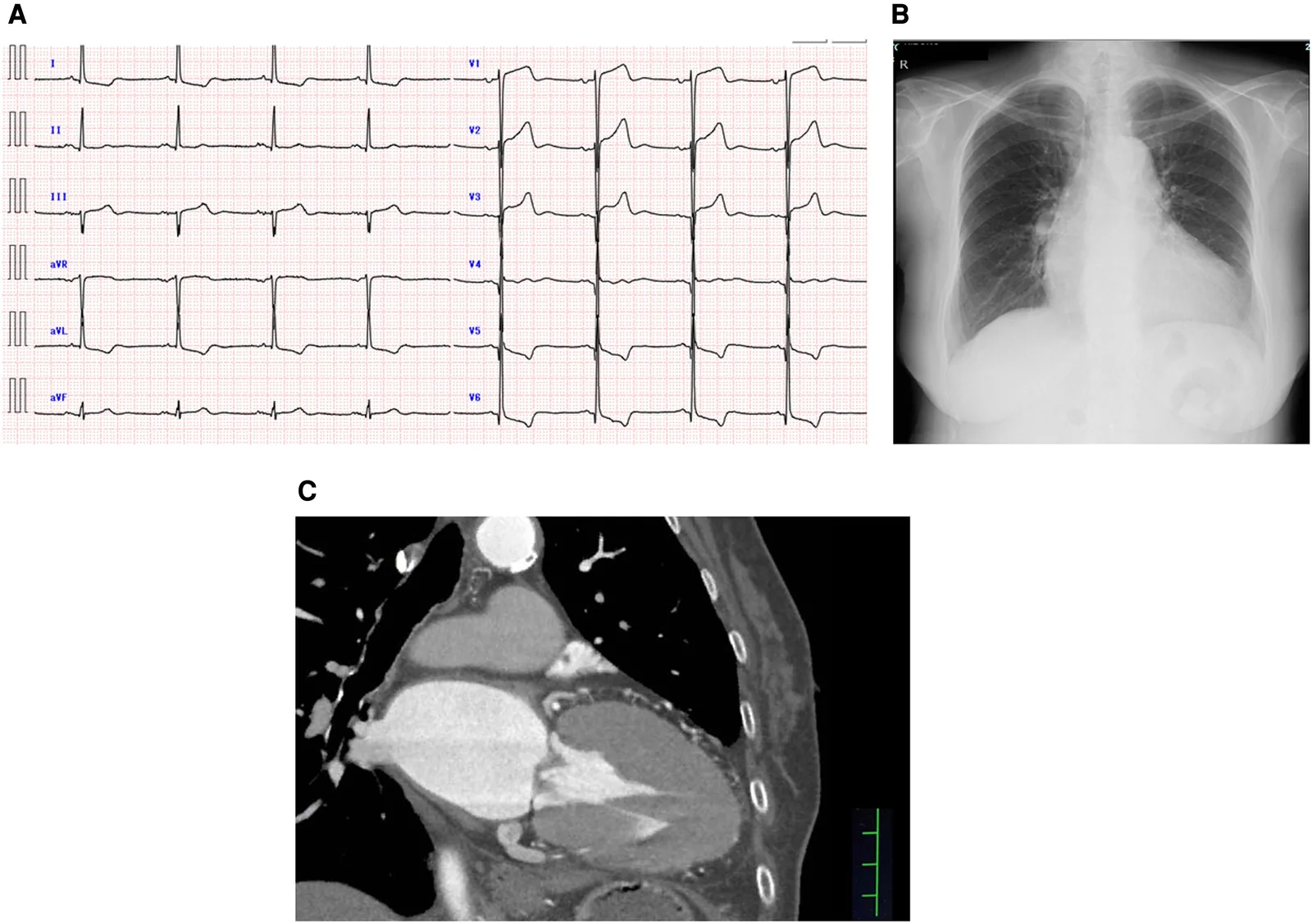
Twelve-lead electrocardiogram (*A*), chest X-ray (*B*), and cardiac computed tomography imaging of the long-axis view at end-diastole (*C*) on admission.

**Figure 2:**
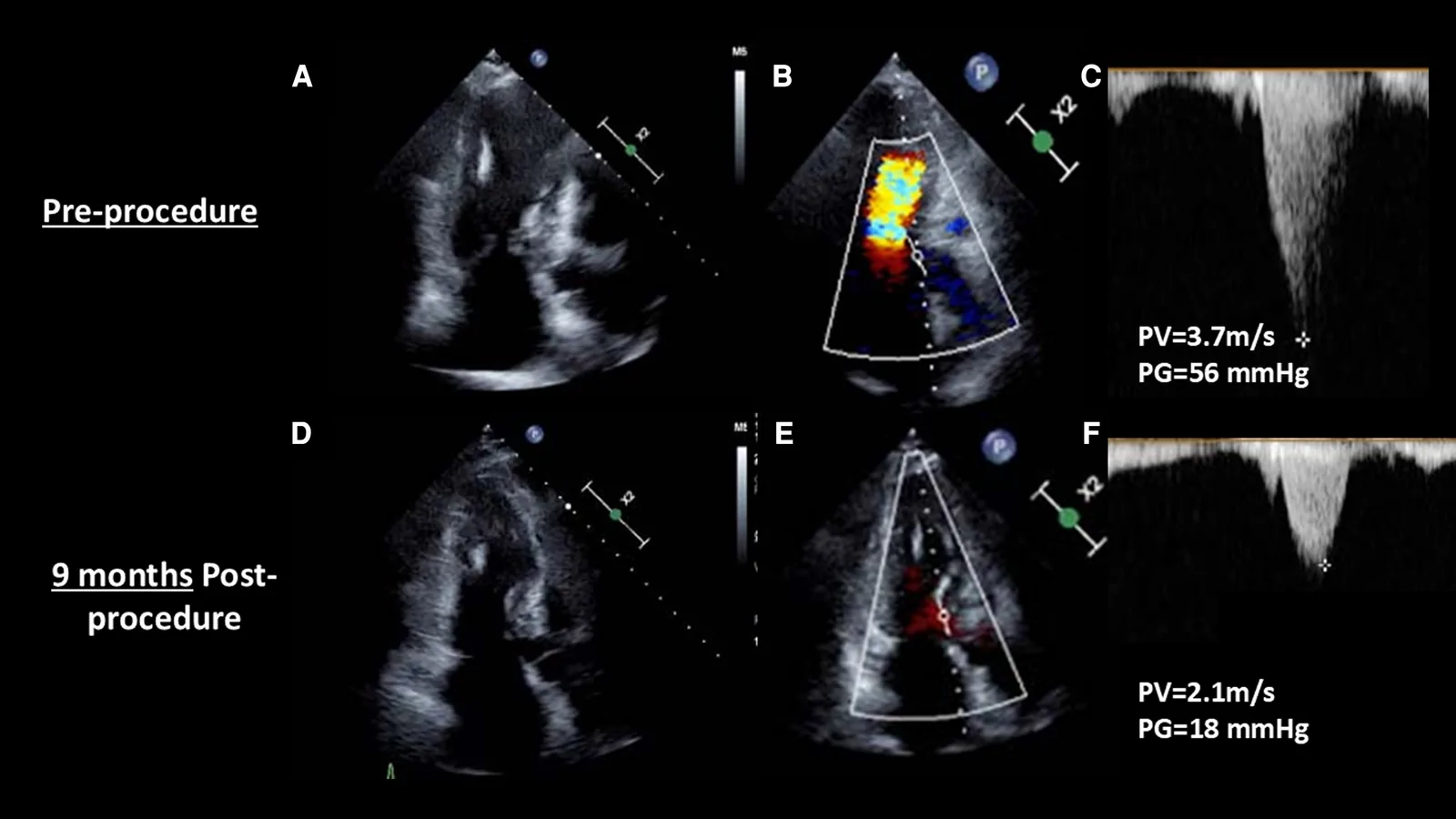
Three-chamber views of B-mode echocardiography (*A* and *D*), Doppler echocardiography (*B* and *E*), and left ventricular outflow tract pressure gradient (*C* and *F*) pre-procedure (*A–C*) and post-procedure (*D–F*). PG, pressure gradient; PV, peak velocity.

**Figure 3:**
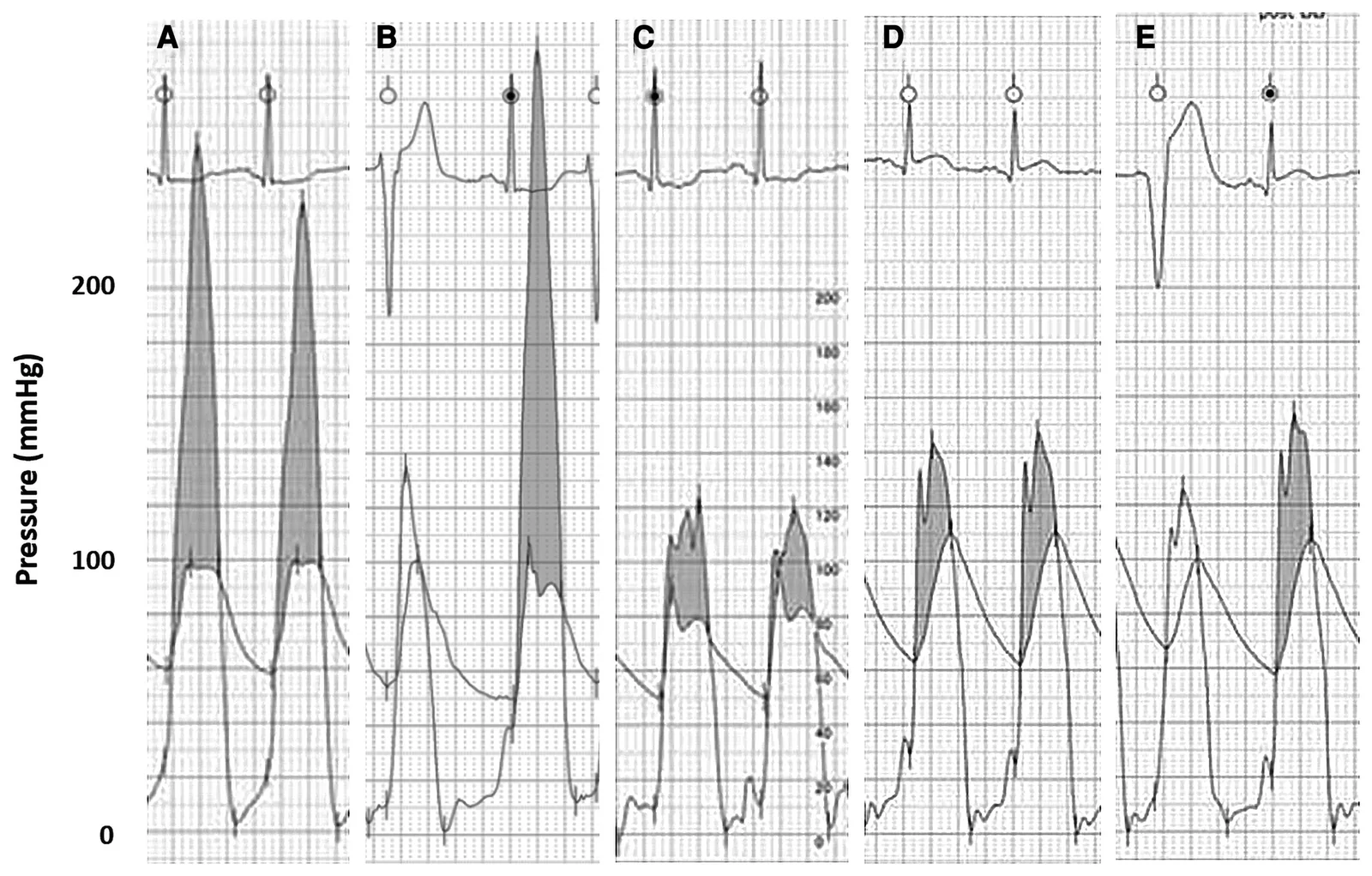
Pressure evaluation at rest (*A*, *C*, and *D*) and post-extra-systolic potentiation (*B* and *E*) between the left ventricular (LV) apex and aorta (*A* and *D*) and at the LV outflow tract (*C*) pre- (*A–C*) and post-procedure (*D* and *E*).

A 4-Fr pigtail catheter inserted via the left femoral artery and a 6-Fr JL3.5 guiding catheter inserted via the right femoral artery were positioned at the LV apex and ostium of the left main trunk, respectively, and simultaneous pressure measurements of the LV apex and aorta were performed. Simultaneous contrast imaging (SCI) with LVG and CAG was performed to evaluate the anatomical relationship between the MVO/LVOTO and the coronary SBs (*Figure 4A and B*; Supplementary material online, *Video S1*). Simultaneous contrast imaging clearly identified one major SB (SB1 in *Figure 4B*) associated with LVOTO and three SBs (SB2–4 in *Figure 4B*) associated with MVO, representing potential PTSMA targets (*Figure 4*). A guidewire (SION^TM^; ASAHI INTECC) was sequentially advanced into the four SBs, and occlusion tests were performed using a 1.5 mm × 8 mm balloon catheter (Emerge OTW^TM^; Boston Scientific). Positive occlusion tests were defined as a >50% decrease in systolic blood pressure during balloon inflation and cold saline injection into the SBs for 10–30 min. The decreases in the gradient with the occlusion of SB1–4 were 60%, 58%, 63%, and 41%, respectively. Three effective target SBs for PTSMA were identified (SB1–3 in *Figure 4B*) and ablated using 1.8, 1.2, and 1.4 mL absolute ethanol in SB1–3, respectively. The total volume was 4.4 mL. Since the patient declined pacemaker implantation, absolute ethanol was injected very slowly (0.1–0.2 mL/min) through the balloon catheter lumen while carefully monitoring the electrocardiogram to avoid the risk of atrioventricular block requiring permanent pacing. Subsequently, the PG between the aorta and LV apex improved to 30–40 mmHg (*Figure 3D*) at rest and 35–45 mmHg (*Figure 3E*) after extra-systolic potentiation. The residual PGs at the LV mid-cavitary level and the LVOT were 10–20 mmHg and 5–15 mmHg, respectively. Final CAG revealed normal left coronary arteries without SBs. The maximum creatine kinase level was 1743 IU/L. The patient was discharged 7 days after the procedure without any complications. At 1-month post-procedure, a cryoballoon (Medtronic) AF ablation was performed (*Figure 5*). At 1-year post-procedure, her NYHA class and BNP level had improved to I and 153.4 pg/mL, respectively. Moreover, SAM (*Figure 2D*) and turbulent flow in the LV (*Figure 2E*) had been abolished, and the maximum PG decreased to 18 mmHg during the Valsalva manoeuvre (*Figure 2F*). Her systolic murmur improved to Levine Grade 1/6, and she remained clinically well after the procedure.

**Figure 4:**
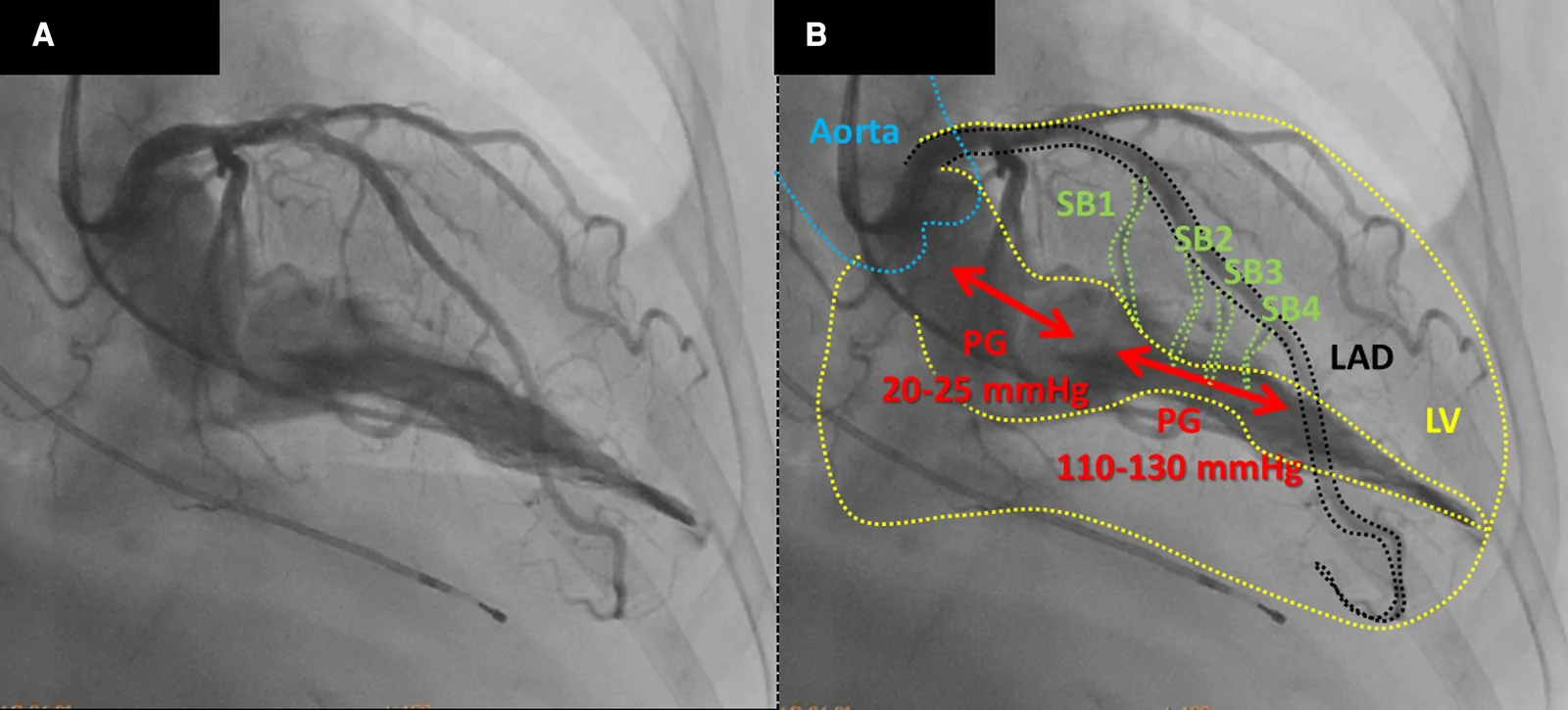
Simultaneous contrast imaging with left ventriculography and coronary angiography (*A* and *B*). LAD, left anterior descending artery; LV, left ventricle; PG, pressure gradient; SB, septal branch.

**Figure 5:**
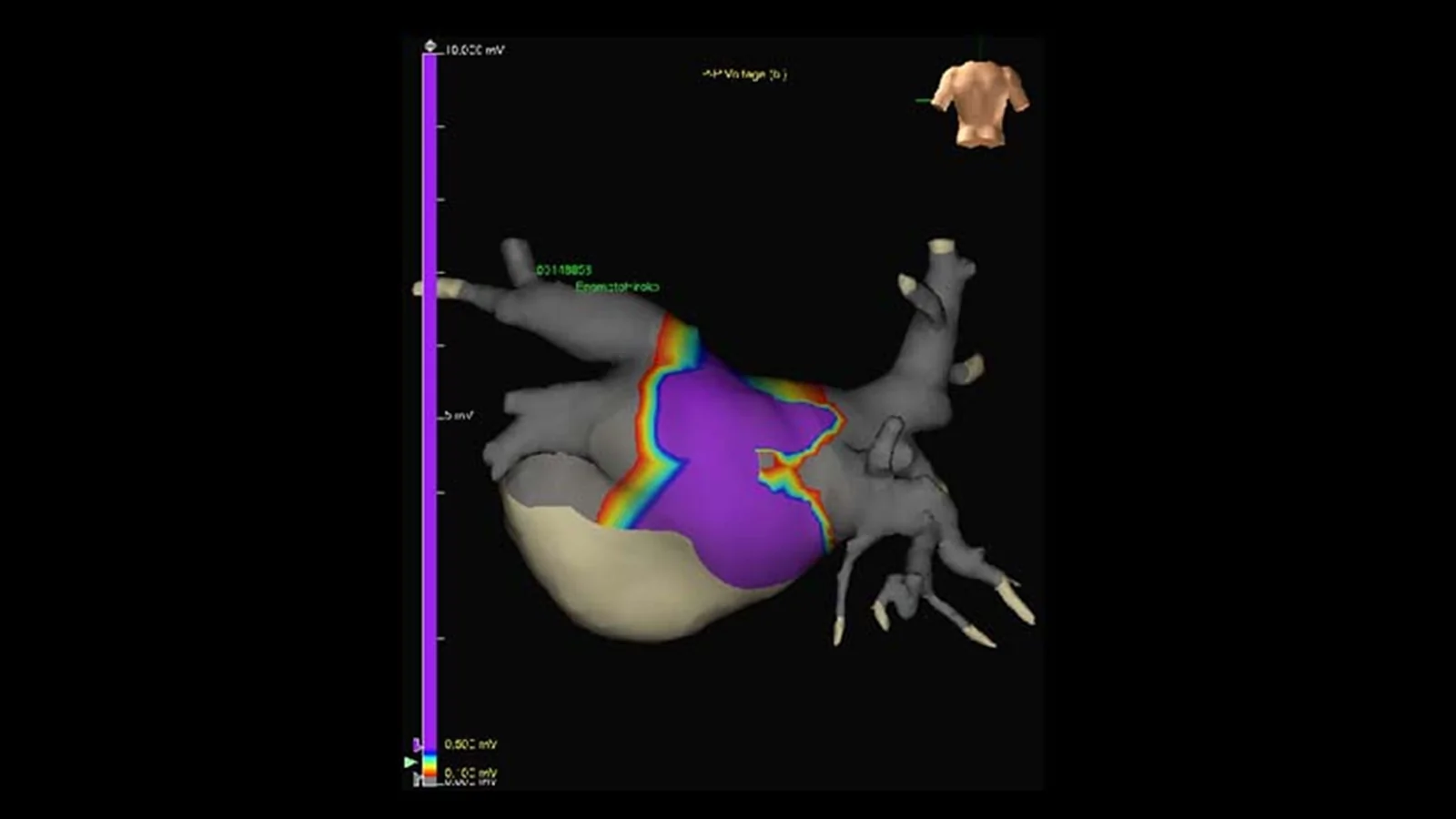
EnSite image after pulmonary vein antrum isolation using a cryoballoon.

## Discussion

Midventricular obstruction is a relatively rare condition characterized by flow impedance at the midventricular level, predominantly caused by marked hypertrophy of the interventricular septum and LV free wall. Midventricular obstruction occurs in ∼5% of American^13^ and 10% of Japanese^1^ patients with HCM. Midventricular obstructions are frequently underdiagnosed or missed, and an unacceptably high proportion of patients may lack optimal treatment.^1^ In this case, based on the findings of turbulent flow in the LV on echocardiography (*Figure 2B*) and the anatomical characteristics on cardiac CT (*Figure 1C*), we suspected MVO and performed detailed LVG and LV interventricular PG evaluations, which resulted in the diagnosis of MVO before the procedure. Considering these findings, an easily applicable resting-state echocardiographic parameter combined with the anatomical characteristics seen on cardiac CT is desirable to discriminate between patients with and without MVO.^14^ However, a recent report showed that cardiac magnetic resonance imaging (MRI) remains a cornerstone in the assessment of hypertrophic cardiomyopathy, allowing evaluation of MVO through morphological, functional, and tissue characterization.^15^ Thus, to avoid potential misinterpretation, the absence of cardiac MRI is a limitation of this case. Previous studies have demonstrated that the presence and severity of MVO are associated with adverse clinical consequences,^16^ and approximately one-fourth of patients with HCM and MVO develop LV apical aneurysms, which are associated with LV thrombus, ventricular arrhythmias, and sudden cardiac death, leading to a poorer prognosis than in patients with LVOTO.^1,17,18^ Generally, patients with MVO have lower LV interventricular PGs than those with LVOTO.^1^ In this case, the PG of the MVO was markedly higher than that of the LVOTO, indicating a high-risk case. Therefore, physicians should consider the possibility of MVO when evaluating patients with HCM to avoid overlooking them.

She declined open-heart surgery^10^ and pacemaker implantation^11^ due to her advanced age (78 years). Previous studies have demonstrated that implantation of a permanent pacemaker was necessary in ∼7%–10% of patients.^3,5^ Therefore, after a shared decision-making process concerning the risk of permanent pacemaker implantation associated with the procedures, the patient accepted it as a bailout treatment. Finally, our heart team concluded that PTSMA should be considered. After providing informed consent, the patient underwent PTSMA.

Percutaneous transluminal septal myocardial ablation for MVO is not well established and may be challenging. A previous report showed that PTSMA for HOCM sometimes resulted in residual obstruction of the midventricular region.^10^ Therefore, before the procedure, we confirmed the detailed anatomical relationship between the MVO/LVOTO and the coronary SBs using SCI with LVG and CAG (*Figure 4A and B*; Supplementary material online, *Video S1*). Furthermore, we identified effective SBs for PTSMA using an occlusion test of the potential target SB1–3, which perfused the area of septal hypertrophy (*Figure 4A and B*; Supplementary material online, *Video S1*) to avoid residual obstruction in the midventricular region. By choosing the optimal SBs, the volume of ethanol infusion can be reduced, and myocardial damage can be minimized, which may reduce periprocedural complications. Systolic anterior motion (*Figure 2D*) was abolished in accordance with the decrease in maximum PG after the procedures. Therefore, mitral valve apparatus abnormalities may have contributed to the outflow gradient in the present case. Finally, favourable clinical outcomes were observed. Thus, confirming and clarifying the detailed anatomical relationship between MVO/LVOTO and effective SBs using SCI with LVG and CAG combined with occlusion tests may be the cornerstone for achieving effective clinical outcomes in patients with HOCM concomitant with MVO. However, future studies are warranted to assess the generalizability of these results.

## Conclusions

Physicians should consider the possibility of MVO when evaluating patients with HCM. Simultaneous contrast imaging with LVG and CAG may be an optional treatment strategy for patients with HOCM and MVO.

## Supplementary Material

ytag659_Supplementary_Data

## Data Availability

The de-identified participant data will not be shared.

## Lead author biography

Dr Togo Sakai received his MD degree from Saga University (Saga, Japan) in 2013. Since 2020, he has been working as a cardiologist at the Social Medical Corporation Steel Memorial Yawata Hospital. His area of medical interest is heart failure, arrhythmias, sleep disorder, cardiac rehabilitation, and coronary artery disease.

## Notes

**Consent:** The authors confirm that the patient provided written consent for the submission and publication of this case report, including the image(s) and associated text, in accordance with the COPE guidelines.
